# Fungal community dynamics associated with the outbreaks of sugarcane root rot disease

**DOI:** 10.1128/spectrum.03090-23

**Published:** 2024-01-08

**Authors:** Qingxiao Ren, Abdullah Khan, Jinxu Zhang, Yixue Bao, Muhammad Tahir Khan, Jihua Wang, Shiqiang Xu, Muqing Zhang

**Affiliations:** 1 Guangxi Key Laboratory of Sugarcane Biology, State Key Laboratory for Conservation and Utilization of Subtropical Agro-Bioresources, Guangxi University, Nanning, China; 2 National Institute for Biotechnology and Genetic Engineering (NIBGE), Faisalabad, Pakistan; 3 Crop Research Institute, Guangdong Academy of Agricultural Sciences, Guangzhou, China; Dominican University New York, Orangeburg, New York, USA

**Keywords:** sugarcane, root rot disease, *Fusarium*, antagonistic fungi, biocontrol

## Abstract

Sugarcane is a critical sugar and bioenergy crop in China. However, numerous factors, including root rot disease, hamper its yield. Root rot disease is a severe agricultural issue, reducing yield and threatening sustainable crop production. The current study aimed to explore the fungal community structure, identify and characterize the primary pathogen for sugarcane root rot in Guangzhou, China. Eighty-nine samples of sugarcane root, stalk, rhizosphere soil, and irrigation water were collected from five sites in Guangzhou, China. Subsequently, 276 fungal strains were isolated to identify the primary pathogens. The five most common genera identified were *Penicillium*, *Fusarium*, *Gongronella*, *Trichoderma*, and *Cladosporium. Fusarium* was more prevalent in the infected soil samples than in healthy ones. Pathogenic assays of the strains revealed that the strain GX4-46 caused 80% of the disease. The strain was confirmed as *Fusarium commune* through phylogenetic and genome sequence analysis. Rhizosphere soil samples from different regional crops were collected to better understand the fungal community structure and the primary pathogen. We observed a significant presence of *Fusarium* in irrigation water, indicating that the root rot disease could originate from the irrigation water and then spread as a soil-borne disease. This research is pioneering and one of the most comprehensive investigations on the occurrence and prevalence of sugarcane root rot disease. This study will serve as a reference for expanding the sugarcane industry and a foundation for further exploration and control of root rot.

**IMPORTANCE:**

Sugarcane, a significant economic crop, faces challenges due to root rot pathogens that accumulate each year in plants and soil through ratoon planting. This disrupts soil microbial balance and greatly impedes sugarcane industry growth. Symptoms range from wilting and yellowing leaves to stunted growth and reduced seedling tillers. The rhizosphere microbiota plays an important role in plant development and soil health. Little is known about root rot fungal community structure, especially in sugarcane. Here, we focused on exploring the main causative pathogen of root rot in the area alongside a detailed survey of the rhizosphere soil of different severity sugarcane cultivars and rotation crops of the region. To validate the findings, we also investigated the irrigation water of the area. Our study revealed *Fusarium commune* as the causative pathogen of root rot in the area, primarily originating from water and later as soil-borne. Using *Trichoderma* can control the disease effectively.

## INTRODUCTION

Sugarcane produces approximately 85% of the global sugar and 40% of the ethanol (1). Sugarcane, a perennial ratooning crop of the family Gramineae, is widely planted in southern China (2). Recent years have witnessed a significant decline in sugarcane growth due to the practice of ratoon planting, resulting in the proliferation of pathogens in both soil and plants that contribute to sugarcane root rot, as well as an ensuing disruption of soil microbial equilibrium. *Fusarium commune*-induced root rot of sugarcane is a significant concern in Guangdong Province, China, particularly in the areas where chewing cane is produced. The disease is characterized by mild-to-severe symptoms. Mild disease manifestation is distinguished by leaf wilting, yellowing, stunted growth, and a reduced tiller count in seedlings. In contrast, severe root rot can cause significant sugarcane mortality and a decrease in crop yield (3).

The complex interaction between plants, the rhizosphere microbiota, and the soil environment is driven by the metabolically active microorganisms in the rhizosphere soil (4). The imbalances in this interaction can have a detrimental impact on plant growth (5). Since microbial pathogens coexist with other microbes in the rhizosphere environment, they experience the same abiotic environmental factors (6). Hence, modifications in microbial physiological activities can affect the overall community structure, impacting the dynamic micro-ecological environment around the root system, which might result in disease development (7). The composition of the rhizosphere microbiota is complex and highly dynamic and is often referred to as the second genome of plants. Research has established a connection between soil-borne diseases and the abundance and behavior of microorganisms within the rhizosphere soil (8). Hence, understanding the structure, function, and interaction of rhizosphere soil microbiota, including the pathogens, is crucial for preventing and controlling soil-borne diseases (9). Studies related to sugarcane microbiota predominantly concentrate on their influence on plant growth and agronomic parameters, with limited attention given to their role in exacerbating sugarcane diseases, such as root rot.

Due to the absence of apparent symptoms in the early stages, there is a lack of adequate preventative methods for sugarcane root rot (3). It is traditionally managed using agricultural or chemical methods; however, these measures are often constrained by geographical limitations, labor unavailability, and seasonal considerations and are occasionally time-consuming (10). Moreover, controlling root rot *via* chemical approaches might result in soil deterioration, microbial drug resistance, and the presence of drug residues in crops (11). In contrast, biological control presents an attractive alternative for treating crop disease due to its ecological friendliness, non-toxicity toward non-target organisms, and lower risk of causing drug resistance in pathogens (12). Harnessing antagonistic microorganisms from rhizosphere soil has been demonstrated as a potential strategy to enhance disease resistance in plants (13). Biocontrol agents possess desirable characteristics, such as rapid reproduction, simple nutritional requirements, excellent adaptability to different environments, and strong root colonization ability (14). For instance, a recent study reported that *Bacillus* species isolated from banana rhizosphere soil could effectively promote the growth of banana seedlings and prevent *Fusarium* wilt disease (15). Biocontrol agents have been isolated from the rhizosphere soil of wheat and corn and have shown inhibitory effects against soil-borne diseases caused by *Fusarium oxysporum*, *Sclerotinia sclerotiorum*, and *Rhizoctonia solani* (16). The control of *Fusarium* from a soil microbial ecology perspective is a globally significant research focus. It has been ascertained that the resistance mechanisms inherent in wheat cultivars resistant to this pathogen may be linked to alterations in the structure of the rhizosphere bacterial community, thereby promoting the colonization of antagonistic microorganisms (6). Furthermore, prolonged, continuous monocropping has been associated with a discernible reduction in the population of culturable soil microorganisms, particularly bacterial taxa, while certain fungal species exhibit an increase in prevalence (16). Therefore, it is imperative to comprehend the intricate interactions between the indigenous microbial community and the invasive pathogens within the soil ecosystem to promote plant health and optimize growth.

The present study was initiated to (i) explore the alteration of fungal communities by evaluating the differences in fungal diversity between healthy and root rot sugarcane samples, including soil, root, and stalk; (ii) explore the fungal diversity of different rotation crops in the region; (iii) identify the primary pathogen causing root rot in sugarcane; and (iv) check the performance and effect of *Trichoderma* as a biological control agent for sugarcane root rot. Irrigation water samples were also collected from the region to identify causative agents in the water. In addition, rhizosphere soil from different rotation crops was collected for analysis. Our investigation highlights the association between the fungal communities and the incidence and severity of sugarcane root rot. Furthermore, the isolation and verification of biocontrol agents hold significant potential for agricultural implementation.

## RESULTS

### Fungal community associated with sugarcane root rot disease

**FIG 1:**
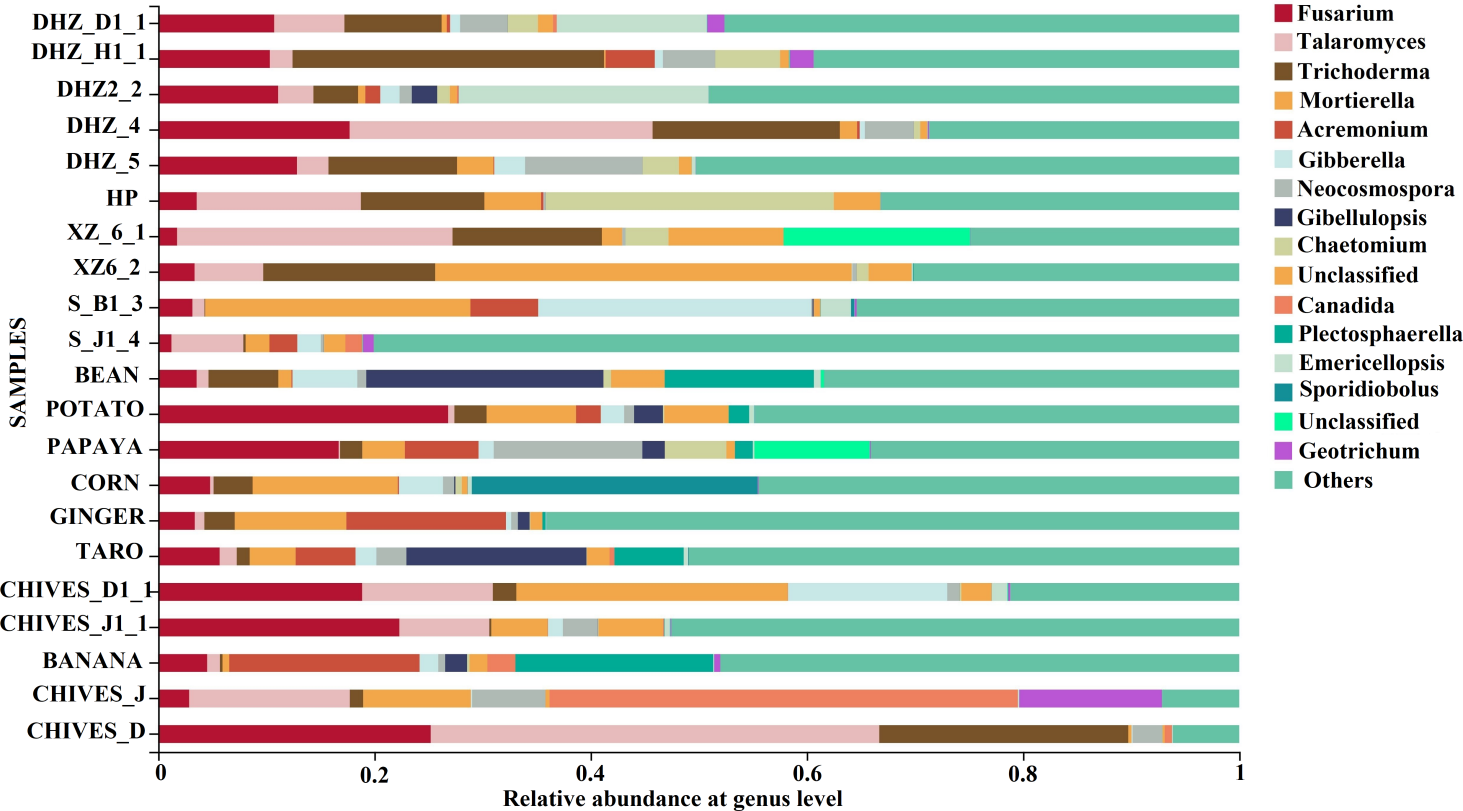
Percent abundance of major fungal genera in the rhizosphere soil of different regional crops. DHZ_D; Susceptible and infected sugarcane, DHZ_H, susceptible and healthy sugarcane; HP, moderately resistant sugarcane; XZ, highly resistant sugarcane; S_D1, soil sample from the non-infected field; S_H1, soil sample from the infected field; Chives_J, healthy sample; Chives_D, infected sample.

**FIG 2:**
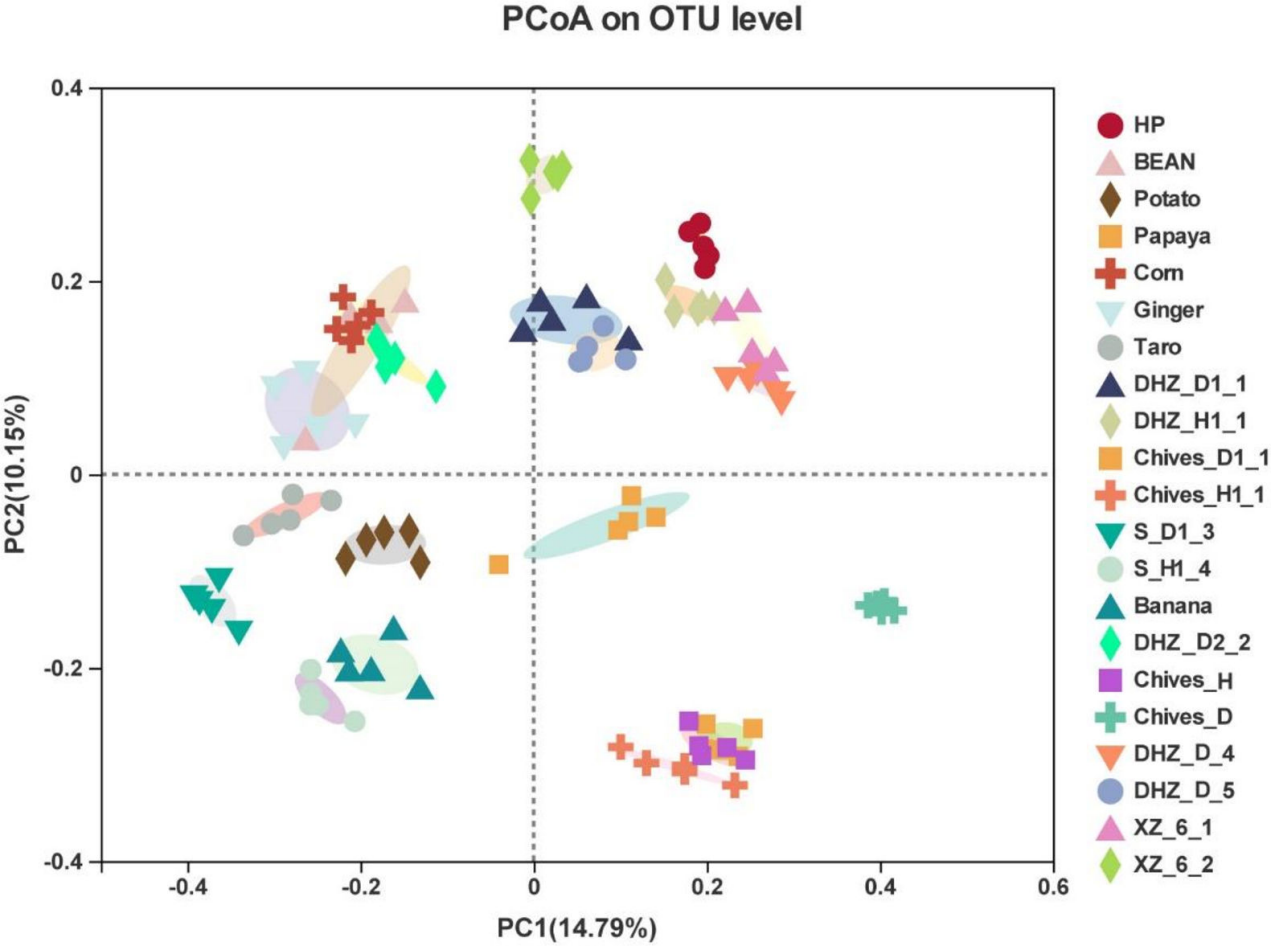
Beta diversity analysis. PCoA of different samples using the Bray-Curtis distance matrix. DHZ_D, susceptible and infected sugarcane; DHZ_H, susceptible and healthy sugarcane; HP, moderately resistant sugarcane; XZ, highly resistant sugarcane; S_D1, soil sample from the non-infected field; S_H1, soil sample from the infected field; Chives_J, healthy sample; Chives_D, infected sample.

Fungal community composition and structure in rotation crops of the root-rot affected area. The rhizosphere soil was sampled, and its microbial diversity was analyzed to better comprehend the growth of different crops in the root rot-affected area and understand the microbial enrichment of this disease in different rotation crops. At the phylum level, the dominant microflora in the soil was mainly composed of Ascomycota, Basidiomycota, Mortierellomycota, and unclassified microorganisms. The details about each crop and the relative abundances of associated significant phyla are illustrated in Fig. S1. The fungal communities were different from the rotation crops at the genus level. The dominant genera in rhizosphere soil were *Fusarium*, *Talaromyces*, *Trichoderma*, *Mortierella*, *Acremonium*, *Gibberella*, *Neocosmospora*, *Gibellulopsis*, and *Chaetomium* (Fig. 1). The relative abundance of *Trichoderma* was higher than that of *Fusarium* in healthy plant soil. *Fusarium* was more prevalent than *Trichoderma* in the disease-affected soils. The overall community of these crops was mainly dominated by *Fusarium* (14.02%), *Talaromyces* (11.46%), *Trichoderma* (10.70%), *Mortierella* (10.38%), and *Acremonium* (4.68%) (Fig. S2). Using the Bray-Curtis distance matrix, PCoA (Principal Coordiante Analysis) analysis was carried out to assess the differences in microbial community composition in the rhizosphere soils of different crops. The separation pattern between samples indicated that different microbial communities had distinct compositions (Fig. 2). Although there were some differences between healthy samples from the same area, the fungal communities of healthy samples from the same crop were similar. Samples were clustered into different groups using hierarchical clustering. The grouping was based on different resistant varieties at each sampling location, indicating that the community structure of various crops was significantly different (Fig. S3).

### Alpha diversity analysis and fungal community structure in different sugarcane cultivars

The diversity indices of Shannon, ACE, Chao1, and Coverage are illustrated in Table 1. The Shannon diversity index of infected sugarcane (DHZ_D) was higher than that of healthy sugarcane (DHZ_H), while its community was affluent. Interestingly, diversity and community richness indices were not evident in root and stalk compared to rhizosphere soil. Nevertheless, the diversity and community richness indices were highest in the soil and stalk compared to the root. The results suggested that DHZ_D exhibited higher fungal diversity and richness than other sugarcane cultivars. The coverage index of all samples was greater than 99%, indicating that the sampling depth was adequate for sequencing (Table 1).

**TABLE 1:** Diversity indices of different sugarcane varieties and samples^*a*^

	Sample	Shannon	Ace	Chao1	Coverage
Soil	DHZ_D	4.12 ± 0.88 a A	717.51 ± 105.8 a A	708.55 ± 105 a A	100%
	DHZ_H	3.62 ± 0.70 a-c A	622.24 ± 98.4 b A	620.46 ± 95 b A	100%
	HP	3.28 ± 0.56 b-d A	450.84 ± 70.2 c A	448.46 ± 82 d A	100%
	XZ	2.88 ± 0.30 cd A	501.38 ± 80.6 c A	467.91 ± 71 c A	100%
Root	DHZ_D	0.95 ± 0.09 fg C	201.28 ± 50.4 e B	185.57 ± 42.1 f B	100%
	DHZ_H	0.52 ± 0.05 g C	180.88 ± 32.4 e-g B	166.27 ± 37.4 g B	100%
	HP	1.55 ± 0.8 ef C	250.14 ± 70.7de B	203.51 ± 22.8 e B	100%
	XZ	1.92 ± 0.8 e C	196.03 ± 31.6 ef B	185.88 ± 38.4 f B	100%
Stem	DHZ_D	2.82 ± 0.9 d B	67.96 ± 11.7 h C	72.33 ± 12.8 j C	100%
	DHZ_H	2.76 ± 0.8 d B	59.06 ± 12.4 h C	54.07 ± 10.6 k C	100%
	HP	3.14 ± 1.1 b-d B	87.04 ± 15.8 gh C	86.11 ± 14.1 i C	100%
	XZ	3.75 ± 0.9 ab B	126.43 ± 22.1 f-h C	146.67 ± 22.8 h C	100%

^*a*^ DHZ_D, susceptible and infected sugarcane; DHZ_H, susceptible and healthy sugarcane; HP, moderately resistant sugarcane; XZ, highly resistant sugarcane. Many samples were compared further to comprehend the genus-level relationship between the non-sample flora. Different lowercase alphabets indicate significant differences between the sugarcane genotypes. Different uppercase alphabets indicate significant differences between soil, root, and stem at least significant difference 0.05.

**FIG 3:**
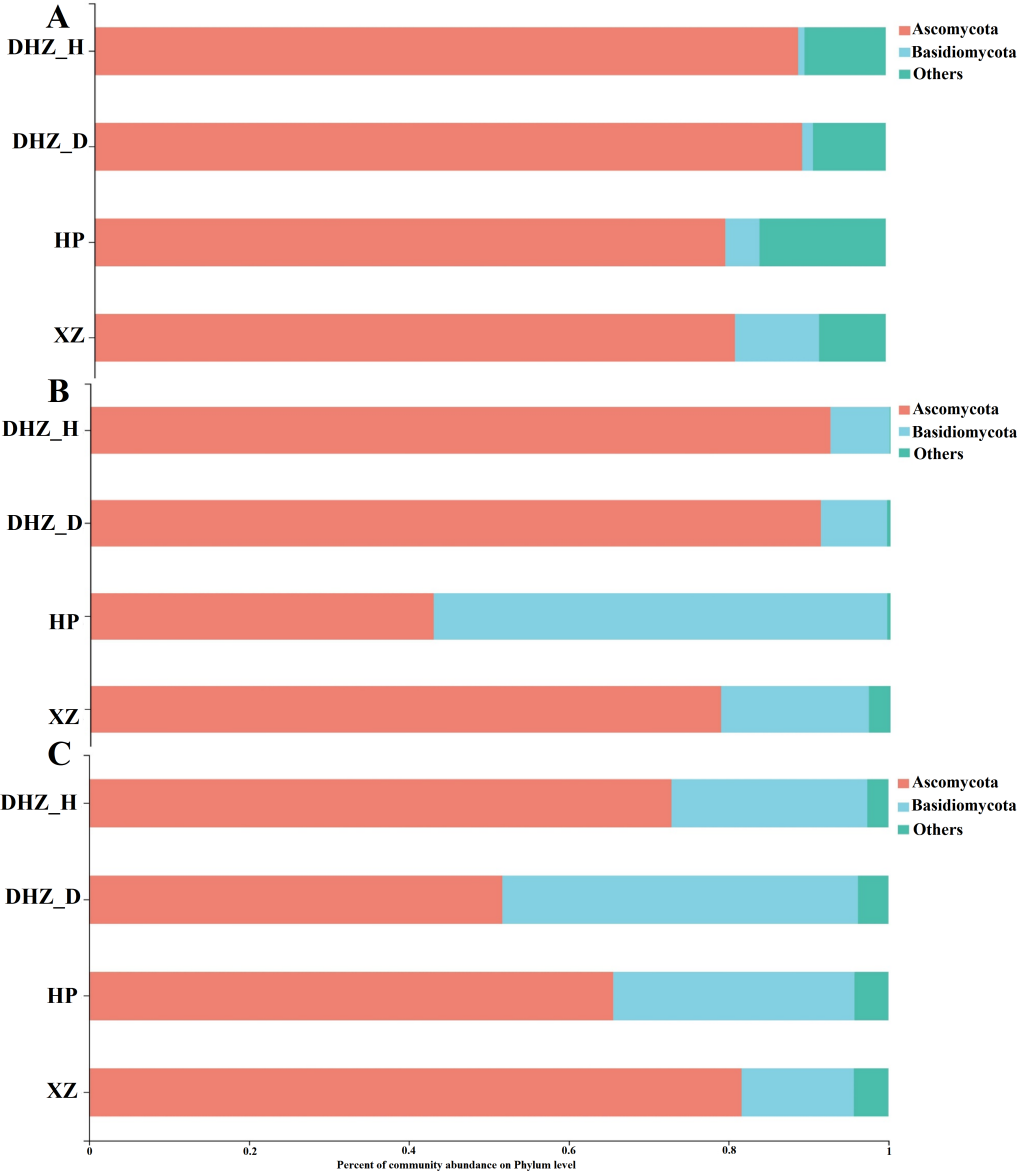
Percent abundance of major fungal phyla. (**A**) Fungal community composition of rhizosphere soil. (**B**) Fungal community composition of sugarcane root. (**C**) Fungal community composition of sugarcane stalks. DHZ_D, susceptible and infected sugarcane; DHZ_H, susceptible and healthy sugarcane; HP, moderately resistant sugarcane; XZ, highly resistant sugarcane.

**FIG 4:**
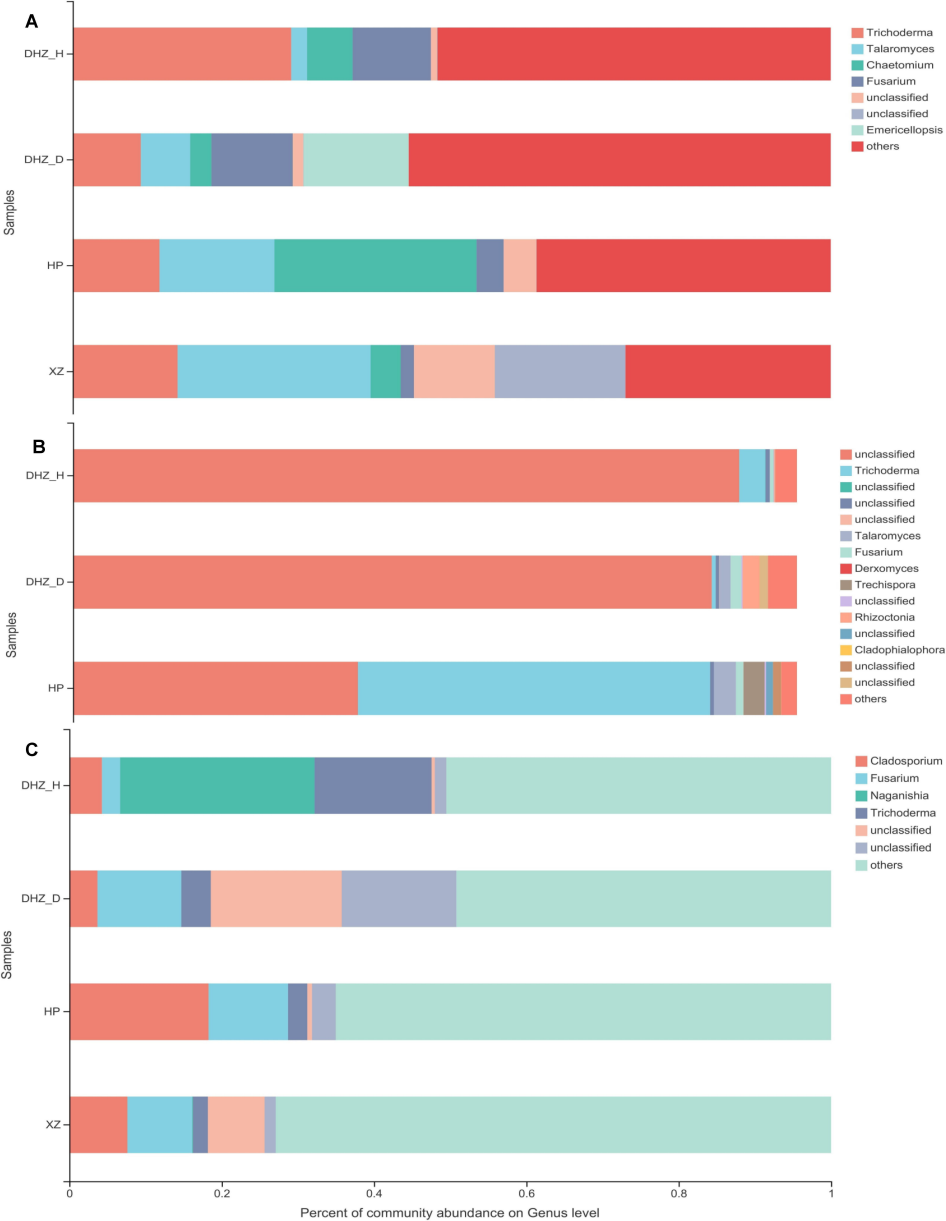
Relative abundance of the fungal community at the genus level. (**A**) Fungal community composition of rhizosphere soil. (**B**) Fungal community composition of sugarcane roots. (**C**) Fungal community composition of sugarcane stalks. DHZ_D, susceptible and infected sugarcane; DHZ_H, susceptible and healthy sugarcane; HP, moderately resistant sugarcane; XZ, highly resistant sugarcane.

Additionally, phyla Ascomycota and Basidiomycota mainly dominated the sugarcane fungal community, while some unclassified communities were observed. Phylum Ascomycota is mainly dominant in the rhizosphere soil, with higher abundance in susceptible sugarcane varieties than in moderate and highly resistant varieties (Fig. 3A). In roots, Basidiomycota was predominant, followed by Ascomycota (Fig. 3B). However, there were variations in the abundance of the dominant fungal phyla in the root compartment. The susceptible varieties had a greater relative abundance of Basidiomycota than the moderately and highly resistant varieties. The Ascomycota was less abundant in the healthy sugarcane stem samples than in the infected, moderately, and highly resistant ones (Fig. 3C). These samples were dominated by *Trichoderma*, *Talaromyces*, *Fusarium*, and *Chaetomium* at the genus level (Fig. 4). In detail, the rhizosphere soil samples of DHZ_H were dominated by *Trichoderma*. On the contrary, *Trichoderma* was less prevalent in DHZ_D. *Fusarium* was more prevalent in the infected sugarcane rhizosphere than in the other rhizospheres. Moderately resistant sugarcane (HP) and highly resistant sugarcane (XZ) showed a higher abundance of *Talaromyces* and a lesser abundance of *Fusarium* (Fig. 4A). Root samples from DHZ_H primarily contained unclassified genera. However, *Trichoderma* was also observed in substantial abundance. DHZ_D contained more *Fusarium* species than DHZ_H in stem samples. Similarly, DHZ_D contained relatively more *Fusarium* than other genera (Fig. 4B). In contrast to the rhizosphere soil and roots, more Cladosporium was present in the HP and XZ stem samples (Fig. 4C).

**FIG 5:**
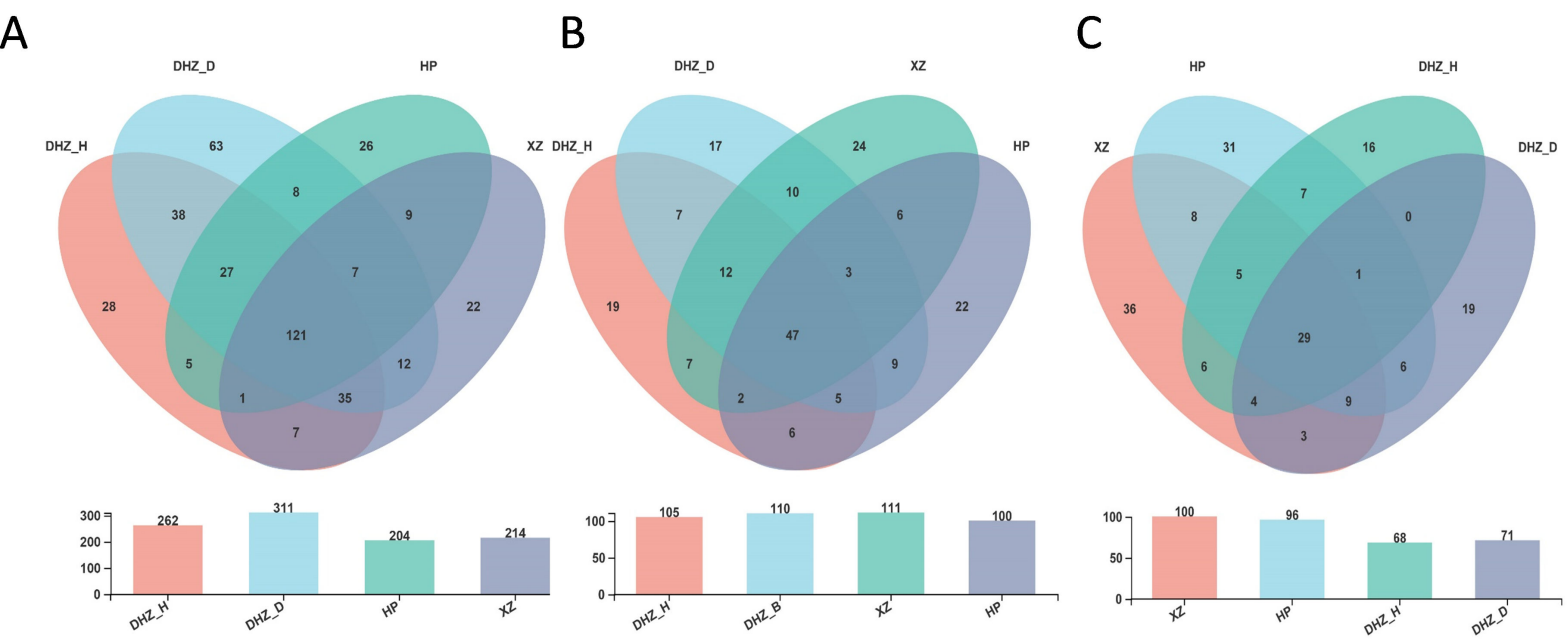
Venn diagram illustrating the number of unique and shared genera in each sample group of different sugarcane parts. (**A**) Number of shared and unique genera in rhizosphere soil. (**B**) Number of shared and unique genera in roots. (**C**) Number of shared and unique genera in the stalk.

A total of 121 genera shared by all four sugarcane types were identified in the rhizosphere soil samples. In contrast, there were 28 unique genera in DHZ_H, 63 in DHZ_D, 26 in HP, and 22 in XZ (Fig. 5A), including 17.60% *Trichoderma,* 11.41% *Talaromyces*, 9.76% *Chaetomium*, and 7.40% *Fusarium* (Fig. S4a). While 47 genera were shared among the analyzed samples, distinct genera were observed, with 17 unique to DHZ_H, 19 to DHZ_D, 24 to HP, and 22 to XZ (Fig. 5B). Most of the root fungal community contained unclassified genera; however, substantial proportions of *Trichoderma* (14.90%), *Talaromyces* (2.52%), and *Fusarium* (1.06%) were also observed (Fig. S4b). Twenty-nine genera were shared among the stem samples. In comparison, 36 genera were found to be unique to XZ, 31 to HP, 16 to DHZ_H, and 19 to DHZ_D (Fig. 5C), including 12.62% *Cladosporium,* 11.59% *Fusarium*, and 9.42% *Trichoderma* (Fig. S4c).

### Beta diversity analysis of different severity sugarcane cultivars

**FIG 6:**
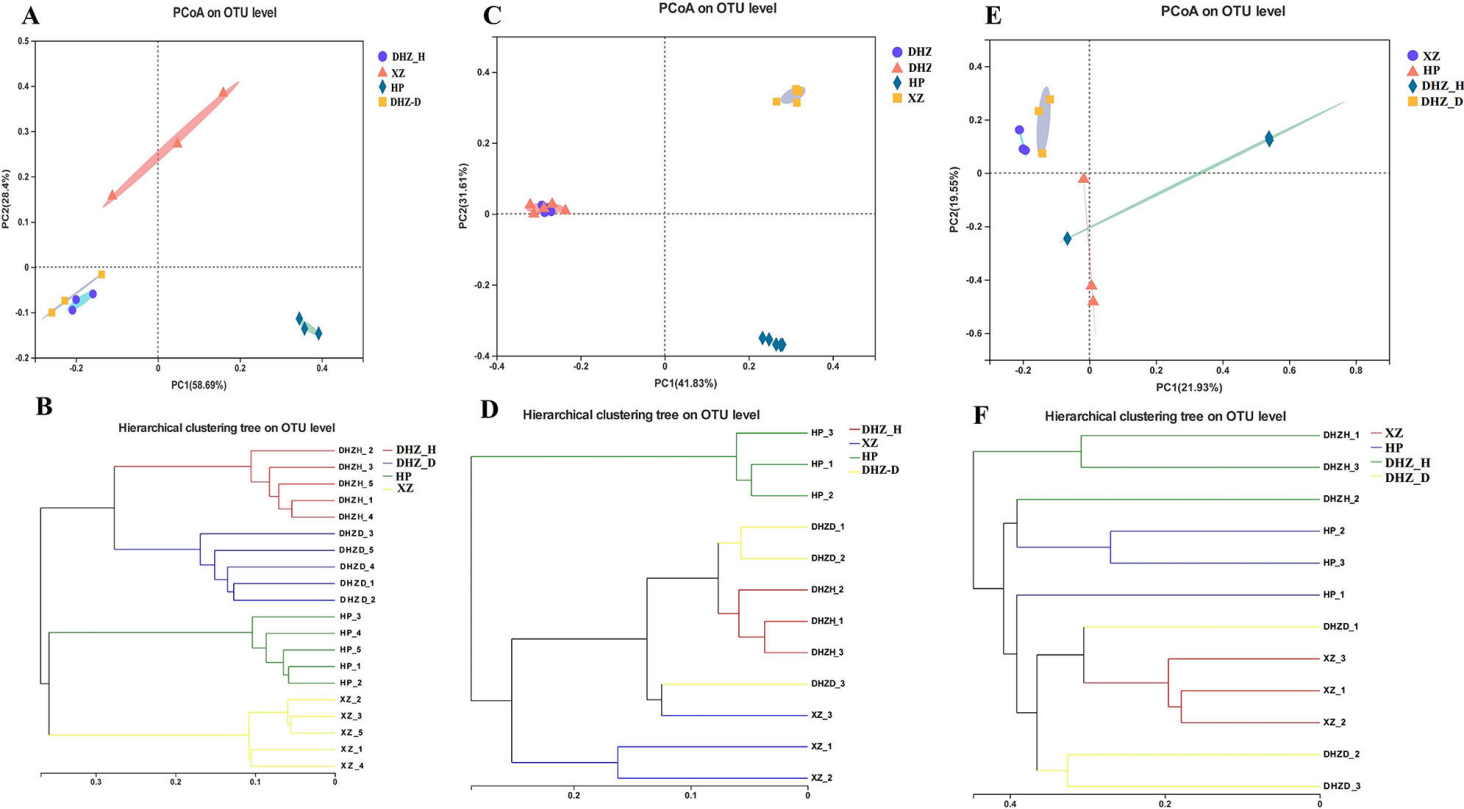
Beta diversity analysis of sugarcane rhizosphere soil. (**A**) PCoA analysis of different genotypes at the OTU level. (**B**) Hierarchical cluster analysis of rhizosphere soil samples. (**C**) PCoA analysis of root samples at OTU level. (**D**) Hierarchical cluster analysis of root samples. (**E**) PCoA analysis of different samples at the OTU level. (**F**) Hierarchical cluster analysis of stem samples. DHZ_D, susceptible and infected sugarcane; DHZ_H, susceptible and healthy sugarcane; HP, moderately resistant sugarcane; XZ, highly resistant sugarcane.

The Bray-Curtis difference was used to calculate and visualize PCoA plots to assess the microbial community differences between the samples. The sample separation pattern indicated that the fungal community composition varied among rhizosphere soils (Fig. 6). The PCoA plot explained a 73.44% variation among the samples, indicating that infected (DHZ_D) and healthy (DHZ_H) susceptible sugarcane varieties shared similar compositions. However, the microbial community differed from the two sugarcane varieties of HP and XZ (Fig. 6A). Hierarchical cluster analysis further confirmed the differences in the rhizosphere fungal community among sugarcane samples (Fig. 6B).

The fungal community in roots also showed visible differences in the PCoA plot. The plot explained 87.29% of the variation among the root samples. DHZ_D and DHZ_H clustered on the PCoA plot, indicating similarity in fungal composition. On the other hand, XZ and HP showed differences (Fig. 6C). Hierarchical cluster analysis further confirmed the differences between root samples for different sugarcane varieties (Fig. 6D). The PCoA plot for stem samples explained 41.48% of the variation. Contrary to the soil and root samples, the samples of DHZ_D and XZ clustered together on the plot, indicating similarity in fungal composition, while the DHZ_H and HP clustered differently on the plot (Fig. 6E). Hierarchical cluster analysis confirmed the similarities and differences (Fig. 6F).

### Fungi isolated in the root-rot-affected area

A total of 276 strains were obtained from 89 samples in sugarcane fields, including rhizosphere soil, stalks, roots, and irrigation water. Among them, 87% (33 genera and 239 species) belonged to the Ascomycota, and 13% (33 species and five genera) to the Basidiomycota (Fig. S5a). The most common genera isolated were *Trichoderma*, *Fusarium*, *Phoma*, *Penicillium*, and *Cladosporium* (Fig. S5b). The root contained 96.6% Ascomycota and 3.3*%* Basidiomycota*,* while the stalk contained 100% Ascomycota. Rhizosphere soil samples contained a variety of fungi, including 77.3% Ascomycota and 33.3% Basidiomycota. Likewise, irrigation water samples contained 97.3% Ascomycota and 2.63% Basidiomycota.

Thirty-eight strains were isolated from sugarcane irrigation water (Fig. S5c), with *Fusarium verticillioides* accounting for 87.18% of the total species. A total of 150 strains were isolated from the rhizosphere soil (Fig. S5d), among which *Gongronella butleri* and *Penicillium janthinellum* accounted for 18.67% and 12.09%, respectively. Moreover, 60 strains were isolated from roots (Fig. S5e), with *Trichoderma* spp. and *Fusarium oxysporum* representing 42.62% and 14.75%, respectively. Additionally, 28 strains were isolated from the sugarcane stalk (Fig. S5f), where *Phoma herbarum* and *Trichoderma* spp. accounted for 35.71% and 28.57%, respectively. Figure S6 displays fungal isolates such as *Fusarium*, *Trichoderma*, and other genera.

### Pathogenicity test

**FIG 7:**
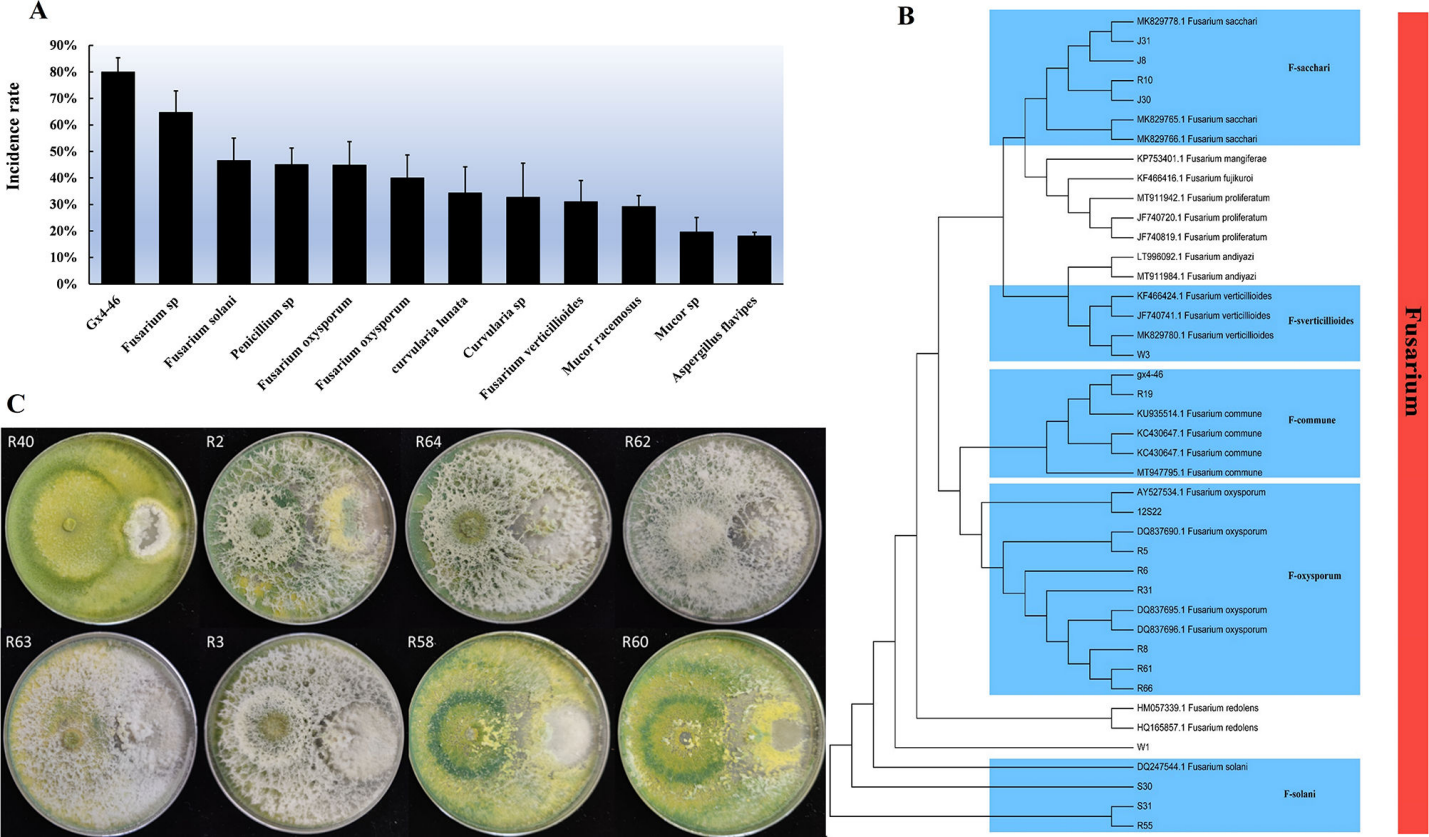
**(A**) Pathogenicity assay of different isolated strains and their disease incidence rate. (**B**) Phylogenetic tree generated from maximum likelihood analysis (RAxML) based on *Fusarium* combined TEF sequence data. Maximum likelihood bootstrap supports values greater than 75%, and Bayesian posterior probabilities greater than 0.95 are indicated on the branches. (**C**) Plate confrontation test of *Trichoderma* against different strains of *Fusarium* performed on potato dextrose agar medium. The strain on the left is *GXU-46*, and the strain on the right is *Trichoderma*.

The spore suspension of potentially pathogenic strains was prepared and inoculated into healthy sugarcane roots for pathogen identification in the greenhouse. Following inoculation, the roots of sugarcane exhibited varying degrees of disease. The disease was characterized by stunted plants, slow growth, gradually dark brown taproots with varying degrees of decay, and a decrease in new roots, similar to the disease in the field. The pathogenicity varied among the different sugarcane root strains. After inoculation with the GX4-46 strain, the incidence in sugarcane roots was up to 80%, significantly higher than other strains. The incidence rates for other strains ranged from 18% to 47% (Fig. 7A). Hence, the GX4-46 strain exhibited the highest incidence rate, and the phylogenetic analysis (Fig. 7B) confirmed that the strain belonged to *Fusarium commune*, the causative agent for root rot in the area.

### Antagonistic effects of *Fusarium* and *Trichoderma*

The two-point confrontation culture test was used to test the effect of *Trichoderma* strains against the GX4-46 strain. The results revealed that the inhibition rate of some strains exceeded 65%, while the inhibition rate of others reached 100%. After 5 days of confrontation culture, strains R40 and R62 invaded the bacterial colony, causing the mycelia to disintegrate and a large area to atrophy (Fig. 7C). Strains R58, R60, and R63 invaded the colony and thinned the mycelia of the junction bacteria. Strains R2 and R3 covered the mycelia of pathogens, inhibiting their growth and preventing their expansion. The bacteriostatic effect of strain R64 was not as pronounced as that of other strains, but it also reduced mycelium growth (Fig. 7C).

## DISCUSSION

The root rot disease is a widespread problem that causes significant crop losses and impacts food security (17, 18). The disease-causing pathogen thrives under plant stress conditions such as monoculture, soil compaction, high soil moisture, and optimal temperature (19). In this study, conventional pathogen isolation and identification methods were combined with high-throughput sequencing techniques to better understand the fungal community structure in the affected crop’s soil and tissue samples. The research focused on identifying the specific root rot pathogen in the Guangdong area and determining its prevention method through biological control. In this research, a fungal strain, GX4-46, was isolated and verified as a causative pathogen belonging to *F. commune*. The present investigation aligns with previous findings (3). We further found that sugarcane root rot did not significantly change the fungal community structure in the area of sugarcane and the rotation crops. Previously, Solis-García et al. (20) found similar results regarding the microbial structure of root-rot-affected avocados. However, the sugarcane cultivars with varying resistance exhibited a change in composition. For instance, the fungal community composition of healthy plants differed from that of infected ones.

It is interesting and noteworthy that infected samples and rotation crops had *Fusarium* in abundance, suggesting that crop rotation might not be a better alternative for controlling the root rot disease. Based on our observations, it could be inferred that *Fusarium* species possess a competitive advantage over other species in the community, which enables them to become dominant during the progression of the disease. It is noteworthy to mention that soil bacterial communities are also closely related to an abundance of *Fusarium* in many crops. Continuous monocropping of a particular crop results in a discernible reduction of the culturable soil bacterial population, while certain fungal species exhibit an increase in prevalence. This phenomenon has been reported for *Fusarium* root rot in multiple crops (21). The findings of fungal communities in diseased and healthy samples coincided with the *Panax notoginseng* root rot (22). The prominent flora of infected and healthy sugarcane varieties changed significantly. Our results were supported by the findings of Yao and Wu (23) and An et al. (24) regarding the microbial community composition of different resistant and susceptible plant species. Another noteworthy result of this experiment was the isolation of *Fusarium* species from the region’s irrigation water. *Fusarium* sp. accounted for more than 90% of the species in the water samples. These results hinted that root rot may be caused by irrigation water in this area. In this regard, future research is advised on more irrigation water precautions. To our knowledge, this research is a pioneering comprehensive study on fungal community structure in various rotation crops and sugarcane cultivars with varying resistance levels against root rot disease.

Biocontrol agents, such as *Trichoderma*, are gaining increasing attention as an alternative to chemical pesticides for controlling plant diseases in sugarcane crops. Despite sugarcane being an important crop worldwide, it is susceptible to various diseases, including fungal infections like root rot. In the present study, *Trichoderma* was present in all samples and was significantly more prevalent in healthy and resistant sugarcane than in susceptible sugarcane. Therefore, *Trichoderma* may antagonistically explain the relationship between root rot incidence in healthy and resistant varieties. *Trichoderma* prevents and controls soil-borne diseases of various vegetables, trees, and maize, such as gray mold, downy mildew, powdery mildew, rice blast, and wheat sheath blight (25). The biological control of *Trichoderma* is managed through competition, hyperparasitism, antibiosis, and the induction of plant resistance. *Trichoderma brevicompactum* has also been extensively used in crop root rot prevention and control. *Trichoderma harzianum* has an excellent antagonistic effect against *Fusarium oxysporium* in maize (26). As a biological control agent, *Trichoderma* has been reported to increase *Rhizoctonia solani* resistance in rice cultivars (27, 28). It antagonistically affects the sugarcane root rot pathogen. *Trichoderma* and *Fusarium* exhibit similar relationships in this region’s rhizosphere soil for different cultivars. *Trichoderma* was prevalent in healthy samples, whereas *Fusarium* was more prevalent in diseased samples. We selected the most pathogenic strains for biological controls to explore the antagonistic effects of *Fusarium* and *Trichoderma*. The plate test results revealed that some *Trichoderma* strains had a strong inhibitory effect on *Fusarium*. The results obtained were on par with the previous report of Filizola et al. (29), who evaluated *Trichoderma* as a biocontrol agent against *Fusarium* in melon.

Furthermore, *Trichoderma harzianum* has been reported to antagonize strawberry botrytis cinerea (30). In addition to their biocontrol activity, *Trichoderma* species can promote sugarcane plants' growth and yield. Applying *Trichoderma harzianum* to sugarcane seedlings improved plant growth and biomass production (31). In another report, applying *Trichoderma viride* to sugarcane cuttings increased the number of tillers, stalk height, and yield (32). However, the effectiveness of *Trichoderma* as a biological control agent in sugarcane crops depends on various factors, such as soil conditions, pathogen level, and application timing. Hence, the abovementioned factors should be considered when utilizing *Trichoderma* in sugarcane fields.

### Conclusion

In this study, we delved into fungal diversity within root rot-affected areas in Guangzhou, aiming to unravel the fungal community structure. *Fusarium* strain GX4-46 was confirmed as causative agent of root rot in the area, and *Trichoderma* as potential biocontrol agent. The implications of our findings extend to plant breeders and pathologists, providing valuable knowledge for comprehending root rot disease and devising effective mitigation strategies. The present study has provided first-hand information on the phylogenetic analysis and the diversity of fungi in the affected area of southern China.

## MATERIALS AND METHODS

### Sampling site and collection

A detailed survey of different sugarcane-growing areas in Guangdong (23.1317° N, 113.2663° E), China, was conducted to collect root-rot-affected and healthy samples. The details of the experimental site and sampling locations are presented in Fig. S7. A total of 75 samples were collected from rhizosphere soil, roots, stalks, and water. Among them, 27 were collected from the rotational crop area abandoned due to cane root rot in preceding years, encompassing a variety of crops, including bean, potato, papaya, corn, ginger, taro, chives, and banana. Additionally, 48 samples were collected from the sugarcane cultivars with different levels of resistance to root rot disease, including susceptible DaHuiZhong (DHZ, a variant of Badila), moderately resistant Huangpi (HP, local table cane from Guangdong), and highly resistant XueZhe (XZ, local table cane from Fujian). The susceptible variety was further classified as DHZ_H (healthy sugarcane) and DHZ_D (infected). The method of sampling described by Khan et al. (33) was used in this study. It involved digging out the roots of each sugarcane plant and manually shaking off any loosely attached soil. The rhizosphere soil, adhering to the roots, was collected from the surface of the roots. The roots were then washed with clean water and left to air-dry. The collected samples were stored in sterile bags for later use.

### Fungal DNA extraction and PCR analysis

Genomic DNA from plant tissue was extracted using SDS (34, 35). DNA from rhizosphere soil was extracted using the FastDNA Spin Kit for Soil following the manufacturer’s instructions (Omega Bio-Tek, Norcross, GA, USA). Then, the quality of the extracted DNA was checked by a Nanodrop Ultrafine Ultraviolet Spectrophotometry Photometer (Eppendorf, USA). The DNA samples for PCR were stored in a −20℃ refrigerator. 18S rRNA gene fragments were amplified using primers ITS1F (5′-CTTGGTCATTTAGAGGAAGTAA-3) and ITS2R (5′-GCTGCGTTCTTCATCGATGC-3′). The PCR amplification was performed following the manufacturer’s instructions. The total reaction volume was 30 µL, including 15 µL MIX (Nanjing Novizan Biotechnology, China), 12 µL ddH_2_O, 0.1 µM of each forward and reverse primer, and 10 ng of genomic DNA. In order to ensure the accuracy of the identification results, the common primers ITS1F and ITS4R were used to identify different strains, and the specific primers of different strains were also used to identify them. PCR products were further sequenced at Shanghai Bioengineering Co., Ltd., Shanghai, China. Sequencing of the V3-V4 regions was performed using Illumina’s Miseq PE300/NovaSeq PE250 platform (Magi Biomedical Technology Co., Ltd., Shanghai, China). The raw data sets generated in this study have been submitted to the NCBI Sequence Read Archive database with a BioProject ID (PRJNA898601).

### Sequence data processing and microbial analysis

Raw tag sequences were verified for quality and merged into clean reads using FLASH software (http://www.cbcb.umd.edu/software/flash, version 1.2.7). Clean reads were assigned to the corresponding sample to obtain valid sequences for each sample. The QIIME (Quantitative Insights into Microbial Ecology v.1.9.0) tool was used to assign the operational taxonomic unit (OTU) of the representative sequences, utilizing the pair-end data as an input file. The OTUs with a 97% similarity threshold were identified using the UCLUST algorithm and the Greengene database as a reference (36). Each OTU sequence represents the taxonomy relative to each sample. Microbiome Analyst was used for analyzing the generated OTU table (37). The incoming data were rarefied to the minimum library size available using the default total sum normalization procedures, while the low variance filtering was set at 20% with a 20% interquartile range. Sequences were filtered with a 20% prevalence in the sample. The relative abundance was used to calculate the unique taxonomy for each sample. Then, the Chao1, ACE, Simpson, and Shannon alpha diversity indices were calculated.

### Culturing and isolation of root rot pathogen and phylogenetic analysis

Samples from root rot-affected plants were collected, and corresponding fungal isolates were obtained by tissue separation using the single spore method (38). Multiple pieces (4 mm^2^) of affected root tissues were cut, disinfected with 75% ethanol for 30 s, and then rinsed three times with ddH_2_O. The fragments were transferred to a potato dextrose agar (PDA) medium after being drained with sterile tissue paper. The fungi from soil samples were separated using the dilution plate technique, while those from irrigation water were isolated using the spread plate technique. The fungal hyphae and spores were examined and categorized using the *Fusarium* laboratory manual. The Koch postulates were used for verification to determine whether they were pathogens. After fungal genomic DNA extraction for molecular identification, the extracted DNA was subjected to PCR amplification following standard protocols (39). Blast search (http://blast.ncbi.nlm.nih.gov/Blast.cgi) was used to identify the pathogen at the genus level, while MEGA7 (https://www.megasoftware.net/) was used to perform phylogenetic analysis.

### Pathogenic assay

After purification, isolated fungal colonies were picked up with a hole punch along the edge of the media plate grown on a PDA plate at 28°C for 5–7 days in darkness or a potato dextrose water liquid culture at 28°C with shaking for 2–3 days. Colonies were gently scraped from the medium with a needle pick, and mycelia were filtered through sterile gauze to obtain spore suspension. The concentration of spore suspension was adjusted to 1.0 × 10^6^/CFU with sterile water. After the canes were sterilized with carbendazim, a healthy root system was developed, and then the buds were grown at 25°C for 7 days. The conidia suspension was uniformly inoculated in the root circumference of healthy sugarcane plants hydroponically. Sterile water was used as the control. Each treatment was repeated three times, and the disease incidence in the plant roots was monitored for 7 days. After the appearance of apparent symptoms, the characteristics and diameter of the disease spots were recorded, and the disease rate was calculated using the following formula (40).


$$Incidence\;(\%)=\frac{number\;of\;infected\;roots}{total\;number\;of\;roots}\times 100$$

### Screening of biocontrol strains

*Trichoderma* and pathogenic fungal agar colonies were cultured for 4 days with a sterilized 6 mm perforator. The prepared *Trichoderma* and pathogenic fungal agar samples were cultured at a 3 mm distance on a PDA culture medium for comparative growth analysis. Each fungal strain and the control were inoculated separately and incubated in the dark at 28°C. Each treatment was replicated three times. The colony diameters of the treatment and control groups were measured by the colony radius calculation method following 5 days of culture. The inhibition rates of each tested strain against phytophthora were calculated according to the following formula:


Colonygrowth(mm)=meancolonydiameter−6.0(mm)

$$\;Inhibition\;(\%)=\frac{total\;single\;colony\;radius-colony\;growth\;radius}{total\;single\;colony\;radius}\times 100$$

### Statistical analysis

A two-way analysis of variance was performed using Statitix 10.0 to indicate significant differences in alpha diversity between the different genotypes and different parts of sampling, e.g., soil, root, and stem.

## Data Availability

The data sets presented in this study can be found in online repositories. The names of the repository(s) and accession number(s) are as follows: https://www.ncbi.nlm.nih.gov/, accession ID: PRJNA898601.

## SUPPLEMENTAL MATERIAL

Fig. S1 spectrum.03090-23-s0001.tif Percent abundance of major fungal phyla in the rhizosphere soil of different regional crops.

Supplemental figure legends spectrum.03090-23-s0002.docx Legends for Fig. S1 to S7.

Fig. S2 spectrum.03090-23-s0003.tif Overall percent abundance of major genera observed in the rhizosphere soil of different crops in the region.

Fig. S3 spectrum.03090-23-s0004.tif Hierarchical cluster analysis of rhizosphere soil samples from different crops in the region.

Fig. S4 spectrum.03090-23-s0005.tif Overall percent abundance of major fungal genera.

Fig. S5 spectrum.03090-23-s0006.tif Relative abundances of fungi isolated from different sources.

Fig. S6 spectrum.03090-23-s0007.tif Different fungal isolates.

Fig. S7 spectrum.03090-23-s0008.tiff Aerial overview of the sampling site.

ASM does not own the copyrights to Supplemental Material that may be linked to, or accessed through, an article. The authors have granted ASM a non-exclusive, world-wide license to publish the Supplemental Material files. Please contact the corresponding author directly for reuse.

## References

[1] Fu Y, Gao H, Yu H, Yang Q, Peng H, Liu P, Li Y, Hu Z, Zhang R, Li J, Qi Z, Wang L, Peng L, Wang Y. 2022. Specific lignin and cellulose depolymerization of sugarcane bagasse for maximum bioethanol production under optimal chemical fertilizer pretreatment with hemicellulose retention and liquid recycling. Renewable Energy 200:1371–1381. doi:10.1016/j.renene.2022.10.049

[2] Khan A, Jiang H, Bu J, Adnan M, Gillani SW, Hussain MA, Zhang M. 2022. Untangling the rhizosphere bacterial community composition and response of soil physiochemical properties to different nitrogen applications in sugarcane field. Front Microbiol 13:856078. doi:10.3389/fmicb.2022.856078 35369493 PMC8964298

[3] Wang J, Chai Z, Bao Y, Wang H, Li Y, Rao GP, Zhang M. 2018. First report of Fusarium commune causing root rot disease of sugarcane (var. Badila) in China. Plant Disease 102:1660. doi:10.1094/PDIS-07-17-1011-PDN

[4] Qu Q, Zhang Z, Peijnenburg W, Liu W, Lu T, Hu B, Chen J, Chen J, Lin Z, Qian H. 2020. Rhizosphere microbiome assembly and its impact on plant growth. J Agric Food Chem 68:5024–5038. doi:10.1021/acs.jafc.0c00073 32255613

[5] Peiffer JA, Spor A, Koren O, Jin Z, Tringe SG, Dangl JL, Buckler ES, Ley RE. 2013. Diversity and heritability of the maize rhizosphere microbiome under field conditions. Proc Natl Acad Sci U S A 110:6548–6553. doi:10.1073/pnas.1302837110 23576752 PMC3631645

[6] Mendes R, Garbeva P, Raaijmakers JM. 2013. The rhizosphere microbiome: significance of plant beneficial, plant pathogenic, and human pathogenic microorganisms. FEMS Microbiol Rev 37:634–663. doi:10.1111/1574-6976.12028 23790204

[7] Xu L, Ravnskov S, Larsen J, Nilsson RH, Nicolaisen M. 2012. Soil fungal community structure along a soil health gradient in pea fields examined using deep amplicon sequencing. Soil Biology and Biochemistry 46:26–32. doi:10.1016/j.soilbio.2011.11.010

[8] Lee S-M, Kong HG, Song GC, Ryu C-M. 2021. Disruption of Firmicutes and Actinobacteria abundance in tomato rhizosphere causes the incidence of bacterial wilt disease. ISME J 15:330–347. doi:10.1038/s41396-020-00785-x 33028974 PMC7852523

[9] Berendsen RL, Pieterse CMJ, Bakker P. 2012. The rhizosphere microbiome and plant health. Trends Plant Sci 17:478–486. doi:10.1016/j.tplants.2012.04.001 22564542

[10] Liu Y, Tian Y, Yue L, Constantine U, Zhao X, Zhou Q, Wang Y, Zhang Y, Chen G, Dun Z, Cui Z, Wang R. 2021. Effectively controlling Fusarium root rot disease of Angelica sinensis and enhancing soil fertility with a novel attapulgite-coated biocontrol agent. Applied Soil Ecology 168:104121. doi:10.1016/j.apsoil.2021.104121

[11] Komárek M, Čadková E, Chrastný V, Bordas F, Bollinger J-C. 2010. Contamination of vineyard soils with fungicides: a review of environmental and toxicological aspects. Environ Int 36:138–151. doi:10.1016/j.envint.2009.10.005 19913914

[12] Tariq M, Khan A, Asif M, Khan F, Ansari T, Shariq M, Siddiqui MA. 2020. Biological control: a sustainable and practical approach for plant disease management. Acta Agriculturae Scandinavica, Section B — Soil & Plant Science 70:507–524. doi:10.1080/09064710.2020.1784262

[13] Mendes R, Kruijt M, de Bruijn I, Dekkers E, van der Voort M, Schneider JHM, Piceno YM, DeSantis TZ, Andersen GL, Bakker P, Raaijmakers JM. 2011. Deciphering the rhizosphere microbiome for disease-suppressive bacteria. Science 332:1097–1100. doi:10.1126/science.1203980 21551032

[14] Chen T, Chen X, Zhang S, Zhu J, Tang B, Wang A, Dong L, Zhang Z, Yu C, Sun Y, Chi L, Chen H, Zhai S, Sun Y, Lan L, Zhang X, Xiao J, Bao Y, Wang Y, Zhang Z, Zhao W. 2021. The genome sequence archive family: toward explosive data growth and diverse data types. Genomics Proteomics Bioinformatics 19:578–583. doi:10.1016/j.gpb.2021.08.001 34400360 PMC9039563

[15] Li W, Peng Z, Yang S, Yu J, Huang J, Wu X, Yang L. 2012. Effects of plant growth-promoting rhizobacteria on growth and controlling Fusarium-wilt disease of banana seedlings. Acta Horticulturae Sinica 39:234–242.

[16] Sun G, Yao T, Liu T, Lu H. 2014. Antagonism of plant growth promoting rhizobacteria on three soil-borne fungus pathogens. Microbiology China:2293–2300. doi:10.13344/j.microbiol.china.140524

[17] Thaines Bodah E. 2017. Root rot diseases in plants: a review of common causal agents and management strategies. ARTOAJ 5:555661. doi:10.19080/ARTOAJ.2017.05.555661

[18] Williamson-Benavides BA, Dhingra A. 2021. Understanding root rot disease in agricultural crops. Horticulturae 7:33. doi:10.3390/horticulturae7020033

[19] Gaulin E, Jacquet C, Bottin A, Dumas B. 2007. Root rot disease of legumes caused by Aphanomyces euteiches. Mol Plant Pathol 8:539–548. doi:10.1111/j.1364-3703.2007.00413.x 20507520

[20] Solís-García IA, Ceballos-Luna O, Cortazar-Murillo EM, Desgarennes D, Garay-Serrano E, Patiño-Conde V, Guevara-Avendaño E, Méndez-Bravo A, Reverchon F. 2020. Phytophthora root rot modifies the composition of the avocado rhizosphere microbiome and increases the abundance of opportunistic fungal pathogens. Front Microbiol 11:574110. doi:10.3389/fmicb.2020.574110 33510714 PMC7835518

[21] Bozoğlu T, Derviş S, Imren M, Amer M, Özdemir F, Paulitz TC, Morgounov A, Dababat AA, Özer G. 2022. Fungal pathogens associated with crown and root rot of wheat in central, Eastern, and southeastern Kazakhstan. J Fungi (Basel) 8:417. doi:10.3390/jof8050417 35628673 PMC9143578

[22] Wu Z, Hao Z, Sun Y, Guo L, Huang L, Zeng Y, Wang Y, Yang L, Chen B. 2016. Comparison on the structure and function of the rhizosphere microbial community between healthy and root-rot Panax notoginseng. Applied Soil Ecology 107:99–107. doi:10.1016/j.apsoil.2016.05.017

[23] Yao H, Wu F. 2010. Soil microbial community structure in cucumber rhizosphere of different resistance cultivars to Fusarium wilt. FEMS Microbiol Ecol 72:456–463. doi:10.1111/j.1574-6941.2010.00859.x 20370829

[24] An M, Zhou X, Wu F, Ma Y, Yang P. 2011. Rhizosphere soil microorganism populations and community structures of different watermelon cultivars with differing resistance to Fusarium oxysporum f. sp. niveum. Can J Microbiol 57:355–365. doi:10.1139/w11-015 21529122

[25] Redda ET, Ma J, Mei J, Li M, Wu B, Jiang X. 2018. Antagonistic potential of different isolates of Trichoderma against Fusarium oxysporum, Rhizoctonia solani, and botrytis cinerea. European Journal of Experimental Biology 8:1–8.

[26] Bharti Y, Vishwakarma S, Kumar A, Singh A, Sharma M, Shukla D. 2012. Physiological and pathological aspects of some new isolates of Colletotrichum falcatum causing red rot disease in Saccharum spp. complex. Acta Phytopathologica et Entomologica Hungarica 47:35-50.

[27] Sharon E, Chet I, Spiegel Y. 2011. Trichoderma as a biological control agent. biological control of plant-parasitic nematodes: building coherence between microbial ecology and molecular mechanisms:183–201. doi:10.1007/978-1-4020-9648-8

[28] Bae S-J, Mohanta TK, Chung JY, Ryu M, Park G, Shim S, Hong S-B, Seo H, Bae D-W, Bae I, Kim J-J, Bae H. 2016. Trichoderma metabolites as biological control agents against phytophthora pathogens. Biological Control 92:128–138. doi:10.1016/j.biocontrol.2015.10.005

[29] Filizola PRB, Luna MAC, de Souza AF, Coelho IL, Laranjeira D, Campos-Takaki GM. 2019. Biodiversity and phylogeny of novel Trichoderma isolates from mangrove sediments and potential of biocontrol against Fusarium strains. Microb Cell Fact 18:89. doi:10.1186/s12934-019-1108-y 31122261 PMC6532204

[30] Roca-Couso R, Flores-Félix JD, Rivas R. 2021. Mechanisms of action of microbial biocontrol agents against botrytis cinerea. J Fungi (Basel) 7:1045. doi:10.3390/jof7121045 34947027 PMC8707566

[31] De Souza JT, Trocoli RO, Monteiro FP. 2016. Plants from the Caatinga biome harbor endophytic Trichoderma species active in the biocontrol of pineapple fusariosis. Biological Control 94:25–32. doi:10.1016/j.biocontrol.2015.12.005

[32] Singh H. 2016. Seed biopriming: a comprehensive approach towards agricultural sustainability. Indian Phytopathol 69:203–209.

[33] Khan A, Wang Z, Chen Z, Bu J, Adnan M, Zhang M. 2021. Investigation of soil nutrients and associated rhizobacterial communities in different sugarcane genotypes in relation to sugar content. Chem Biol Technol Agric 8:1–13. doi:10.1186/s40538-021-00244-5

[34] Quek MC, Chin NL, Tan SW. 2021. Optimum DNA extraction methods for edible bird's nest identification using simple additive weighting technique. Foods 10:1086. doi:10.3390/foods10051086 34068860 PMC8153580

[35] Xia Y, Chen F, Du Y, Liu C, Bu G, Xin Y, Liu B. 2019. A modified SDS-based DNA extraction method from raw soybean. Biosci Rep 39:BSR20182271. doi:10.1042/BSR20182271 30647109 PMC6361772

[36] Flynn JM, Brown EA, Chain FJJ, MacIsaac HJ, Cristescu ME. 2015. Toward accurate molecular identification of species in complex environmental samples: testing the performance of sequence filtering and clustering methods. Ecol Evol 5:2252–2266. doi:10.1002/ece3.1497 26078860 PMC4461425

[37] Khan A, Jiang H, Bu J, Adnan M, Gillani SW, Zhang M. 2022. An insight to rhizosphere bacterial community composition and structure of consecutive winter-initiated sugarcane ratoon crop in Southern China. BMC Plant Biol 22:74. doi:10.1186/s12870-022-03463-6 35183114 PMC8857817

[38] Xiao R, Chen Y, Chen M, Ruan C, Zhu Y, Liu B. 2020. Pathogen identification of root rot of Pseudostellaria heterophylla plant and fungicide screening for its efficient control. Journal of Plant Protection 47:1333–1342.

[39] Herron DA, Wingfield MJ, Wingfield BD, Rodas CA, Marincowitz S, Steenkamp ET. 2015. Novel taxa in the Fusarium fujikuroi species complex from Pinus spp. Stud Mycol 80:131–150. doi:10.1016/j.simyco.2014.12.001 26955193 PMC4779798

[40] Gritton ET. 1990. Registerations of five root rot resistant germplasm lines of processing pea. Crop Science 30:1166–1167. doi:10.2135/cropsci1990.0011183X003000050064x

