# Supplementary figures and images for: Fungal community dynamics associated with the outbreaks of sugarcane root rot disease
Source: Microbiol Spectr. 2024 Jan 8;12(2):e03090-23. doi: 10.1128/spectrum.03090-23 (PMC10845956; doi:10.1128/spectrum.03090-23)

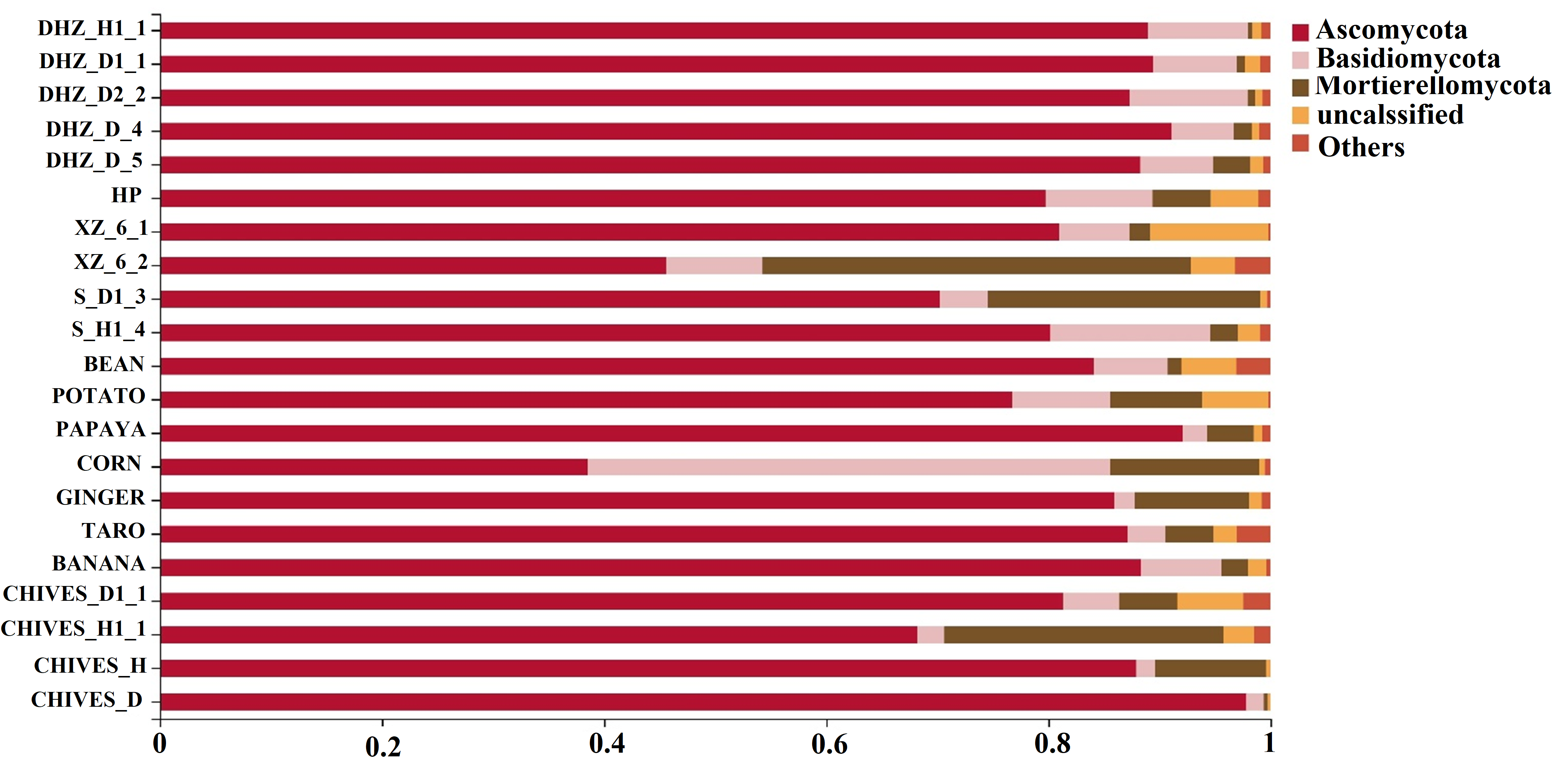

Supplement: Fig. S1 — Percent abundance of major fungal phyla in the rhizosphere soil of different regional crops. [file spectrum.03090-23-s0001.tif]

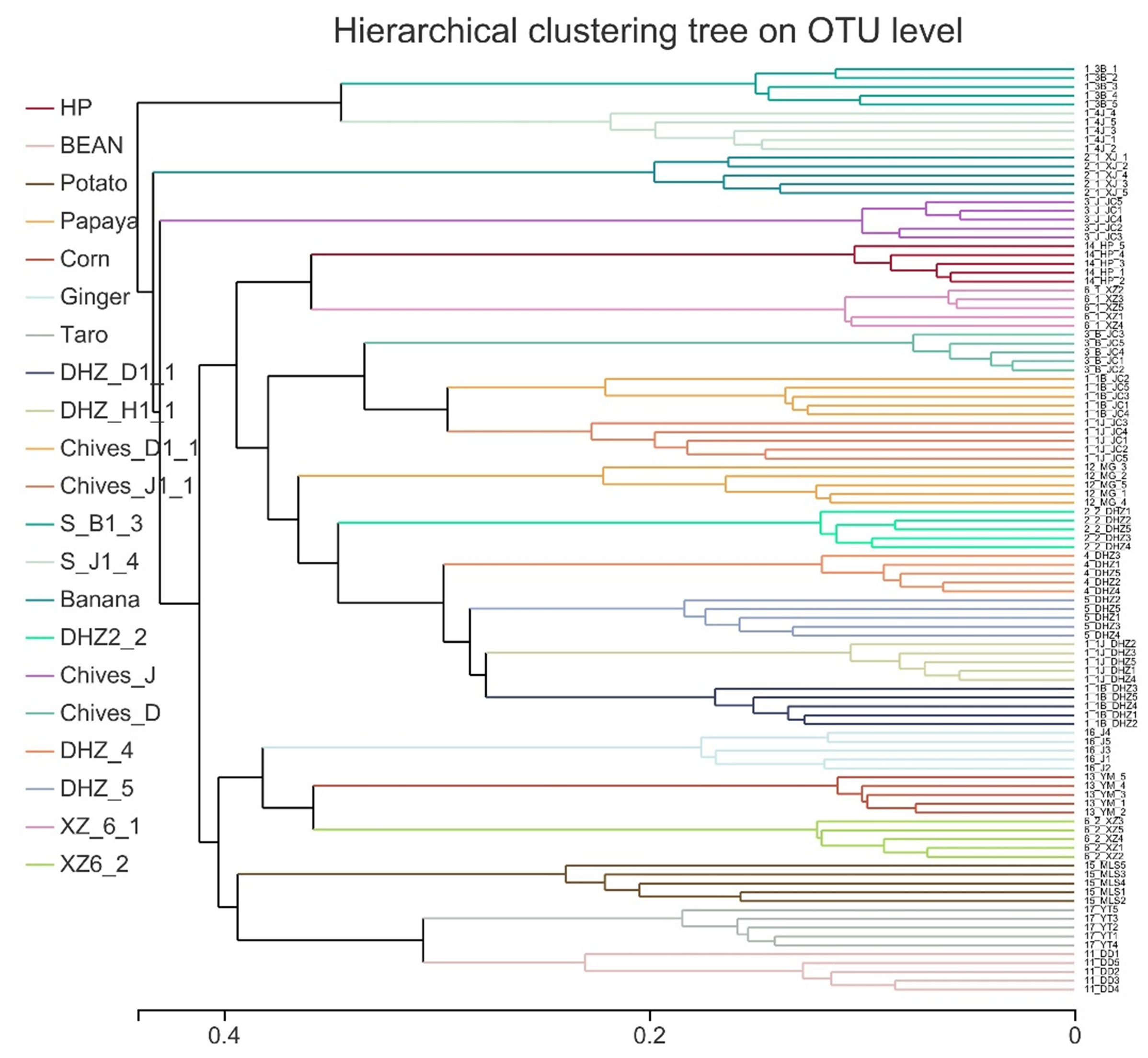

Supplement: Fig. S2 — Overall percent abundance of major genera observed in the rhizosphere soil of different crops in the region. [file spectrum.03090-23-s0003.tif]

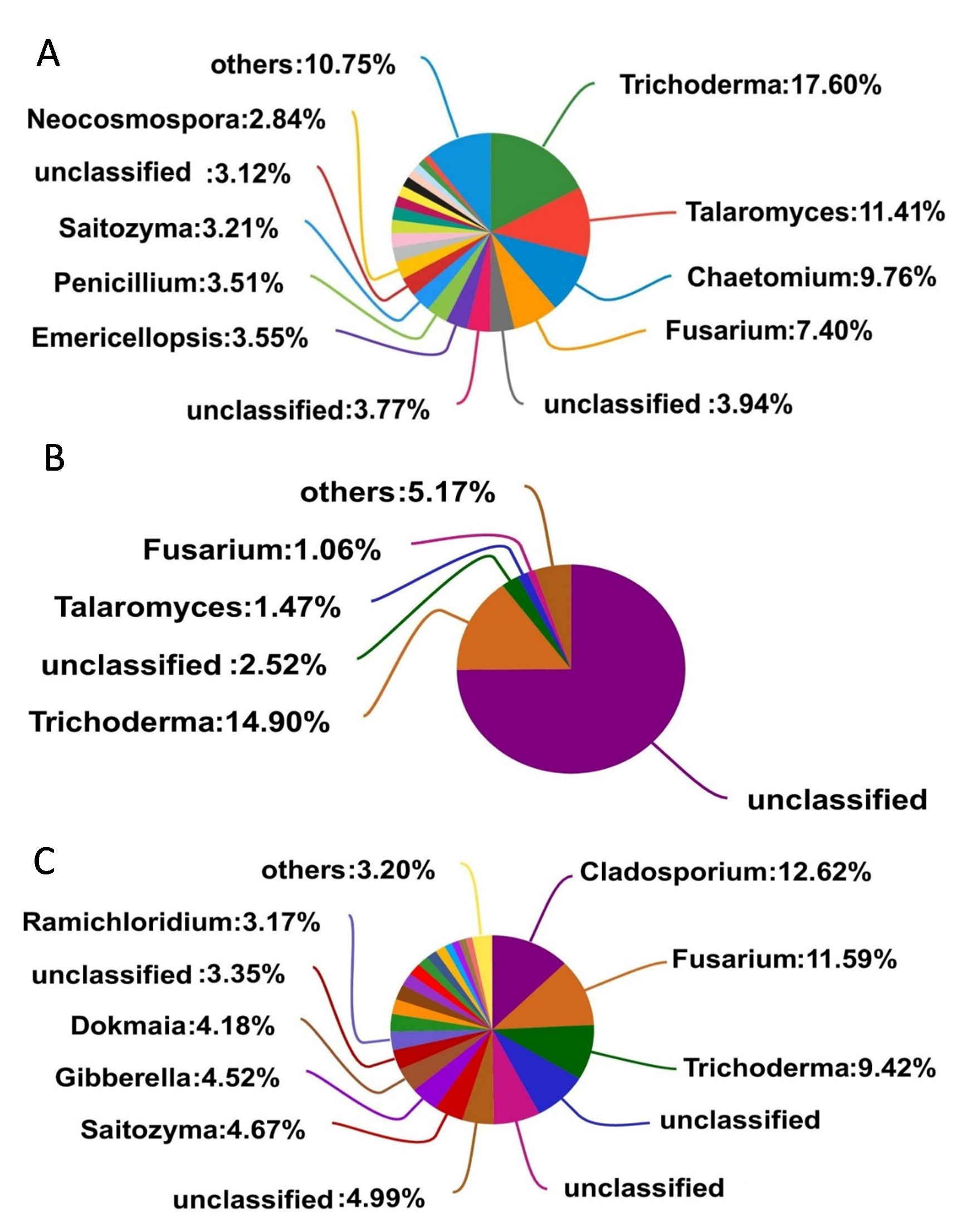

Supplement: Fig. S3 — Hierarchical cluster analysis of rhizosphere soil samples from different crops in the region. [file spectrum.03090-23-s0004.tif]

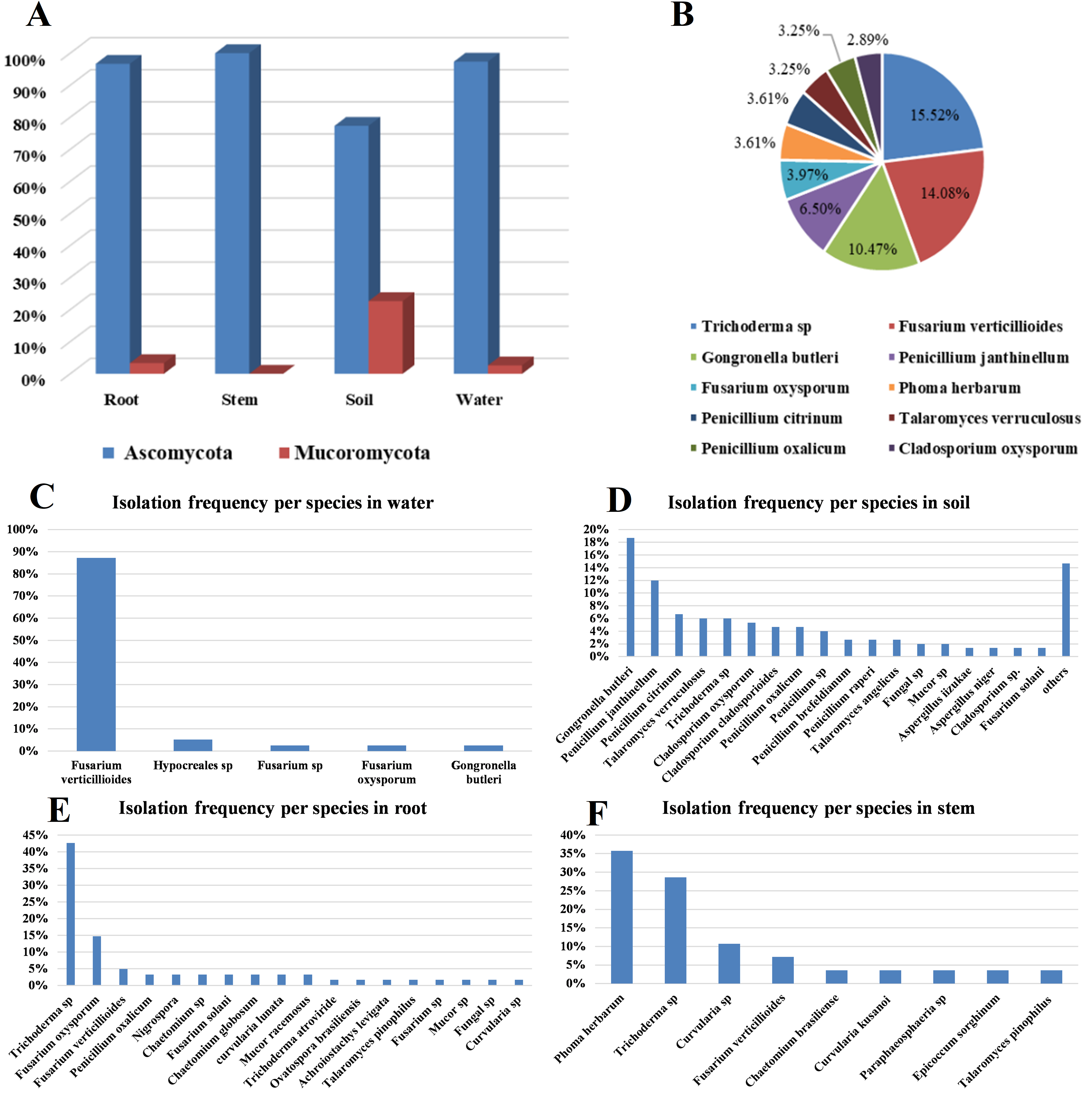

Supplement: Fig. S4 — Overall percent abundance of major fungal genera. [file spectrum.03090-23-s0005.tif]

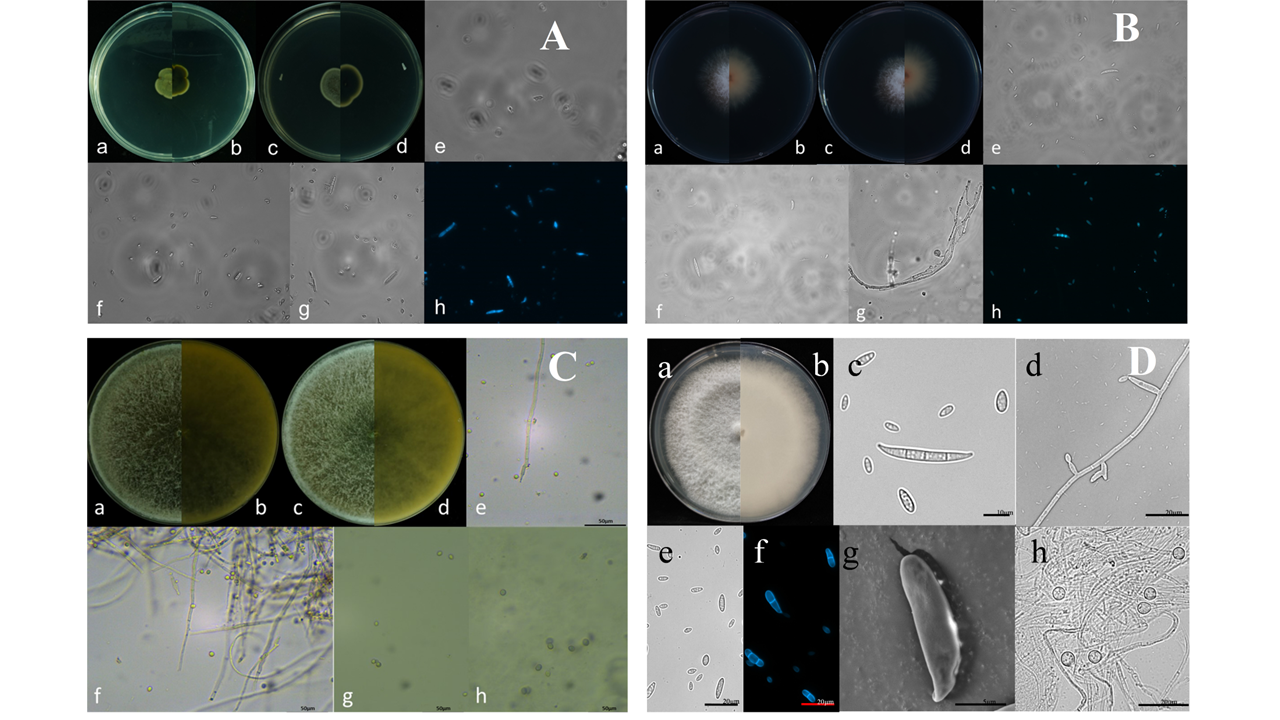

Supplement: Fig. S5 — Relative abundances of fungi isolated from different sources. [file spectrum.03090-23-s0006.tif]

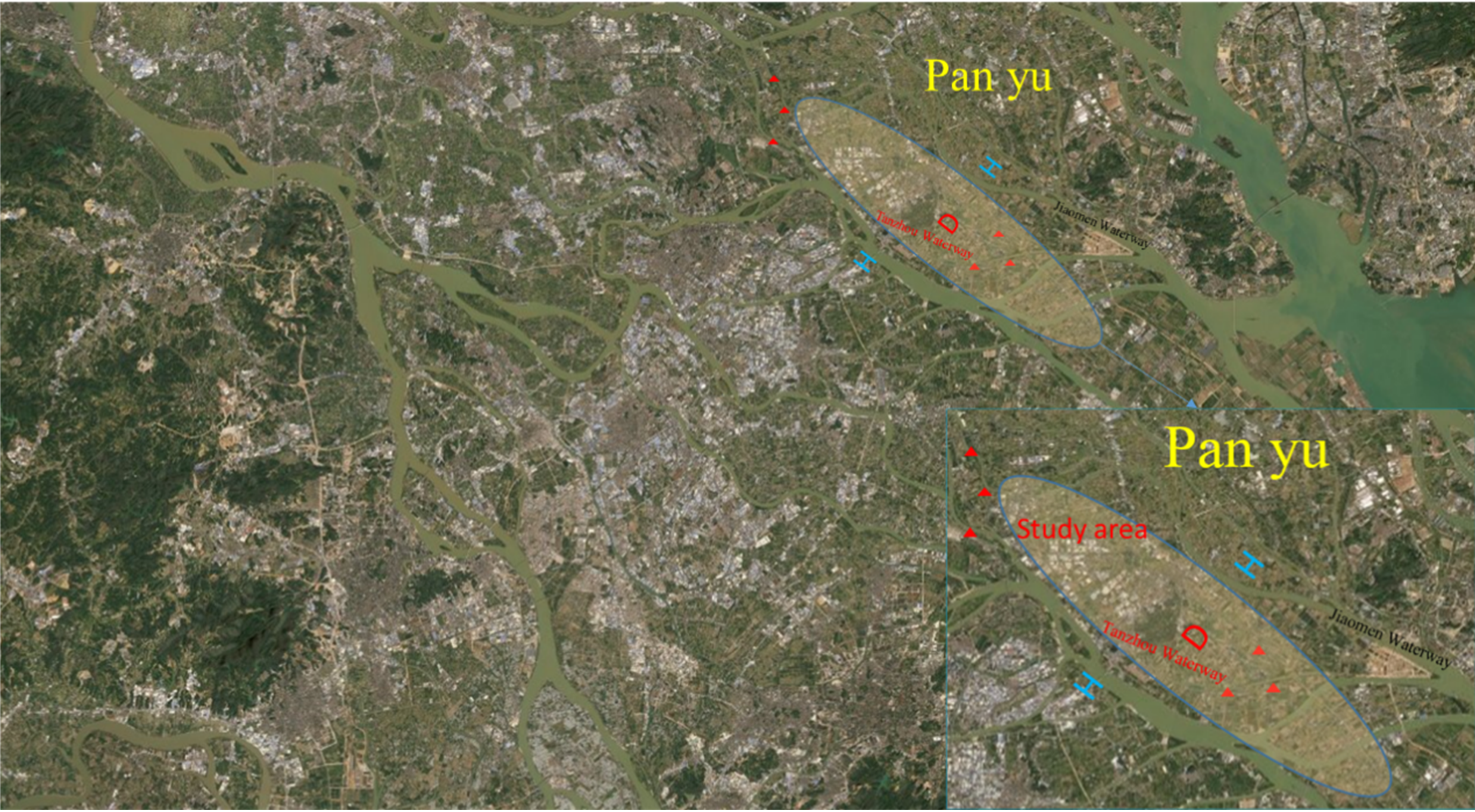

Supplement: Fig. S6 — Different fungal isolates. [file spectrum.03090-23-s0007.tif]

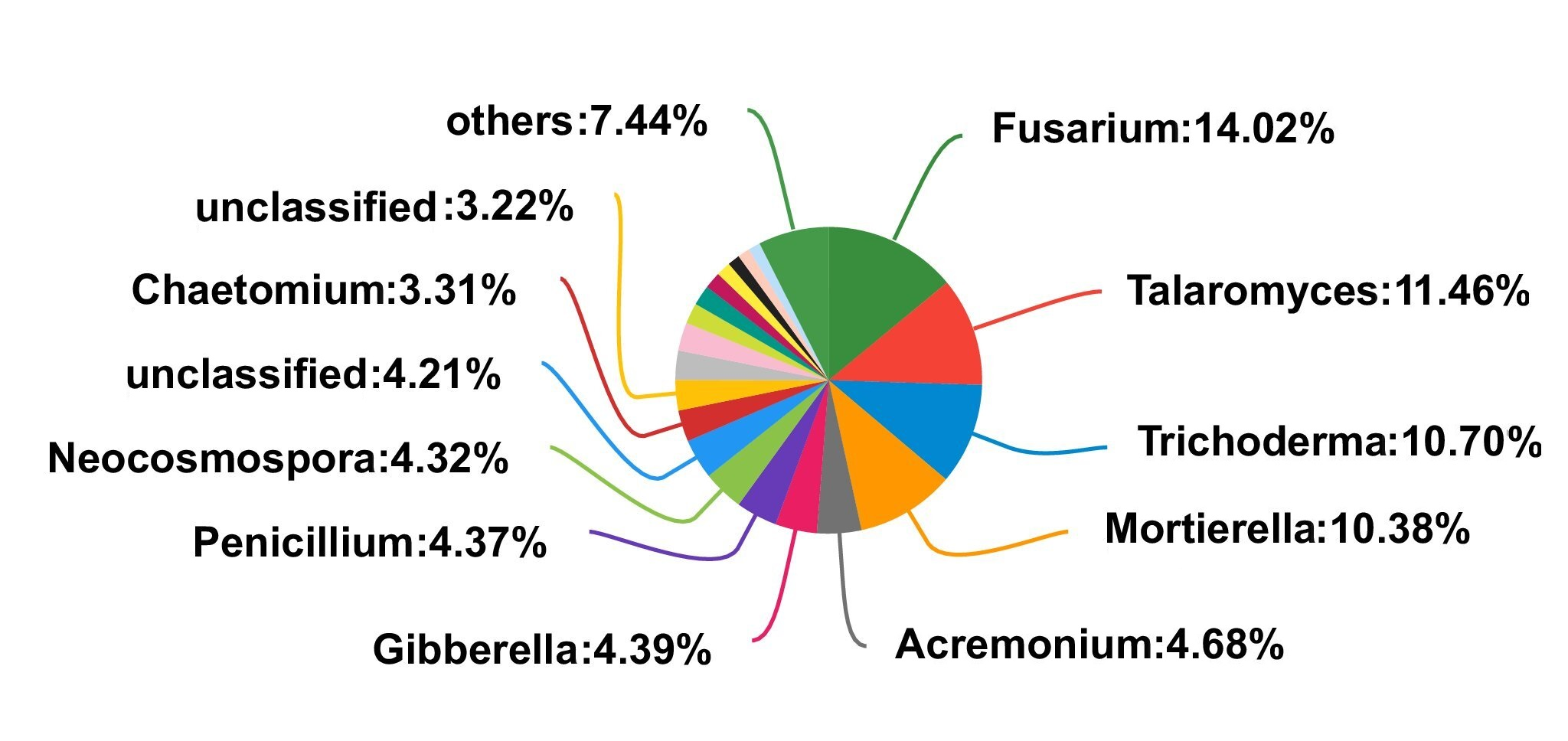

Supplement: Fig. S7 — Aerial overview of the sampling site. [file spectrum.03090-23-s0008.tiff]
