# Supplementary material for: Fungal community dynamics associated with the outbreaks of sugarcane root rot disease
Source: Microbiol Spectr. 2024 Jan 8;12(2):e03090-23. doi: 10.1128/spectrum.03090-23 (PMC10845956; doi:10.1128/spectrum.03090-23)
Supplement: Supplemental figure legends — Legends for Fig. S1 to S7. [file spectrum.03090-23-s0002.docx]

**Supplementary files**

**Figure S1** Percent abundance of major fungal phyla in the rhizosphere soil of different regional crops. DHZ-D; Susceptible and infected sugarcane, DHZ-H; Susceptible and healthy sugarcane, HP; Moderately resistant sugarcane, S_D1; Soil sample from the non-infected field, S_H1; Soil sample from the infected field, XZ; Highly resistant sugarcane. Chives_J; Healthy sample, Chives_D; Infected sample

**Figure S2.** Overall percent abundance of major genera observed in the rhizosphere soil of different crops of the region.

**Figure S3.** Hierarchical cluster analysis of rhizosphere soil samples from different crops of the region. DHZ-D; Susceptible and infected sugarcane, DHZ-H; Susceptible and healthy sugarcane, HP; Moderately resistant sugarcane, XZ; Highly resistant sugarcane. S_B1; Soil sample from the non-infected field, S_J1; Soil sample from the infected field, Chives_J; Healthy sample, Chives_D; Infected sample.

**Figure S4.** Overall percent abundance of major fungal genera. A) percent abundance of fungal genera in rhizosphere soil, B) percent abundance of fungal genera in roots, C) percent abundance of fungal genera in the stem.

**Figure S5.** A) Relative abundance of fungal strains isolated from sugarcane roots, stalk, water, and rhizosphere soil based on ITS sequence. B) abundance of different genera isolated from infected and healthy sugarcane. C) frequency of species isolated from water samples. D) frequency of species isolated from soil samples. E) frequency of species isolated from roots samples. F) frequency of species isolated from stem samples.

**Figure S6.** Different fungal isolates (A) *Cladosporium* (B) *Fusarium* (C) *Trichoderma* and (D) GX4-46 strain. Lower case alphabets ab represents colony from above, cd represents colony from below, e-h represents conidiogenous cells giving rise to conidia.

**Figure S7.** Aerial overview of the sampling site. The inside of the ellipse represents the whole incidence area, △ represents the sampling site, D represents the incidence area, and H represents the non-infected area.
